# When a Benign Tumor Turns Troublesome: A Case Report of Deep Lipoma Compressing the Posterior Interosseous Nerve

**DOI:** 10.1155/cro/5819552

**Published:** 2026-06-01

**Authors:** Pushkar Pudasaini, Lok Raj Chaurasia, Kiran Kishor Nakarmi

**Affiliations:** ^1^ Fellowship in Hand and Microsurgry, Department of Burns Plastics and Reconstructive Surgery, Kiritpur Hospital, Kathmandu, Nepal

**Keywords:** case report, lipoma-induced compression, PIN, posterior interosseous nerve palsy

## Abstract

This case report presents a rare instance of posterior interosseous nerve (PIN) compression caused by a deep lipoma in a patient. The patient was undiagnosed even after visiting multiple doctors and obvious clinical signs were missed. The clinical presentation, diagnostic approach, surgical intervention, and outcome are discussed. This case highlights the importance of considering rare causes of nerve compression and underscores the critical role of clinical examination in guiding diagnosis and management, particularly in resource‐constrained environments. Furthermore, this case also shows that good recovery can be achieved even when there is severe and prolonged compression.

## 1. Introduction

The PIN is a terminal branch of the radial nerve responsible for innervating the wrist and finger extensors [1]. Different sites have been described as possible site of compression of the nerve like leash of Henry at the radial neck, the fibrous edge of the extensor carpi radialis brevis muscle, arcade of Frohse and the distal edge of the supinator muscle [2]. Compression of this nerve can result in weakness of wrist, fingers, and thumb extension. Soft tissue tumors rarely compress peripheral nerve and accounts for only 1.02%–4.9% of cases and compression by a lipoma is even rarer with literature limited to only case reports and case series [3]. This report highlights the case of a patient with PIN compression attributed to a deep lipoma diagnosed and treated in resource‐limited setting.

## 2. Case Report

A 45‐year‐old female presented to the hand clinic with complaints of pain in proximal forearm, weakness in wrist and finger extension on the right side for the past 1 year. She had difficulty in performing daily activities but surprisingly, she was more bothered by pain than weakness of fingers. There was no history of trauma. She had visited other centers where MRI of cervical spine was done, which was unremarkable, and was put on steroids and physiotherapy without any working diagnosis. However, the symptoms worsened so she came to our center for further management.

Upon examination, there was no visible swelling and no obvious asymmetry between bilateral forearms. The patient had weakness in the extension of wrist with radial deviation of the wrist on extension. Her finger and thumb extensors power was zero (MRC grading 0/5). Sensation of the hand was normal. Her elbow extensor power was normal. Imaging study was advised after electrodiagnostic study but due to financial constraints, patient refused further imaging studies.

Nerve conduction study showed Normal SNAP for radial sensory nerve, decreased Extensor Indicis Proprius CMAP and normal Brachioradialis CMAP.

Electromyography showed decreased motor unit potential for Extensor Digitorum Communis and Extensor Pollicis Longus and absent MUP for Abductor Pollicis Longus. The electrodiagnostic report gave diagnosis of axonal injury of PIN at proximal forearm. As the patient had unilateral weakness, which suggested PIN involvement on clinical examination and was also supported by electrodiagnostic studies, provisional diagnosis of PIN compression at proximal forearm was made.

Given that 1 year had elapsed since the onset of symptoms, a discussion was held with the patient regarding surgical options, including immediate tendon transfer versus nerve decompression. After detailed discussion, patient opted for nerve decompression at that time and tendon transfer in future if recovery fails.

### 2.1. Surgical Technique

**Figure 1:**
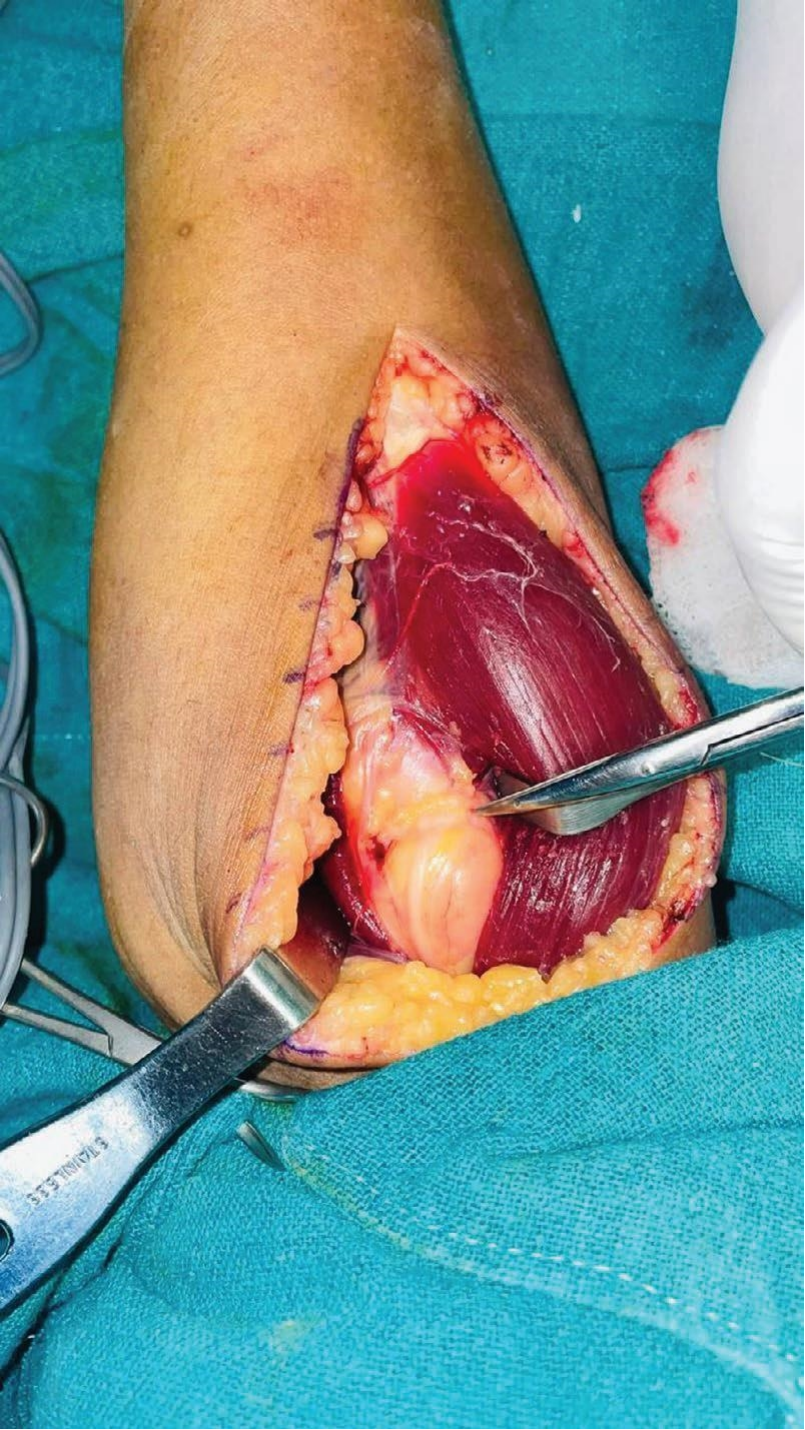
PIN entering arcade of Frohse with lipoma underneath.

**Figure 2:**
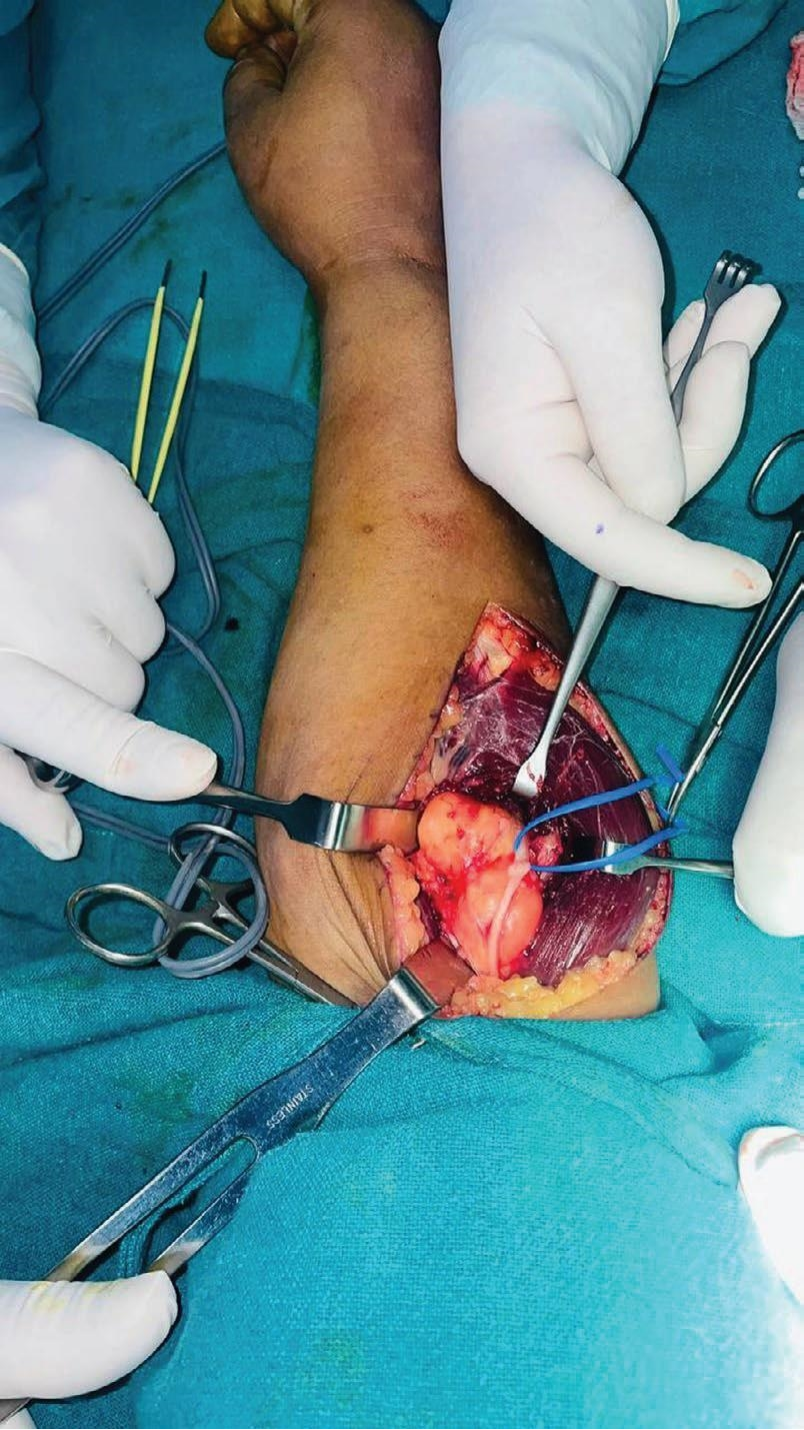
Division of arcade of Frohse.

**Figure 3:**
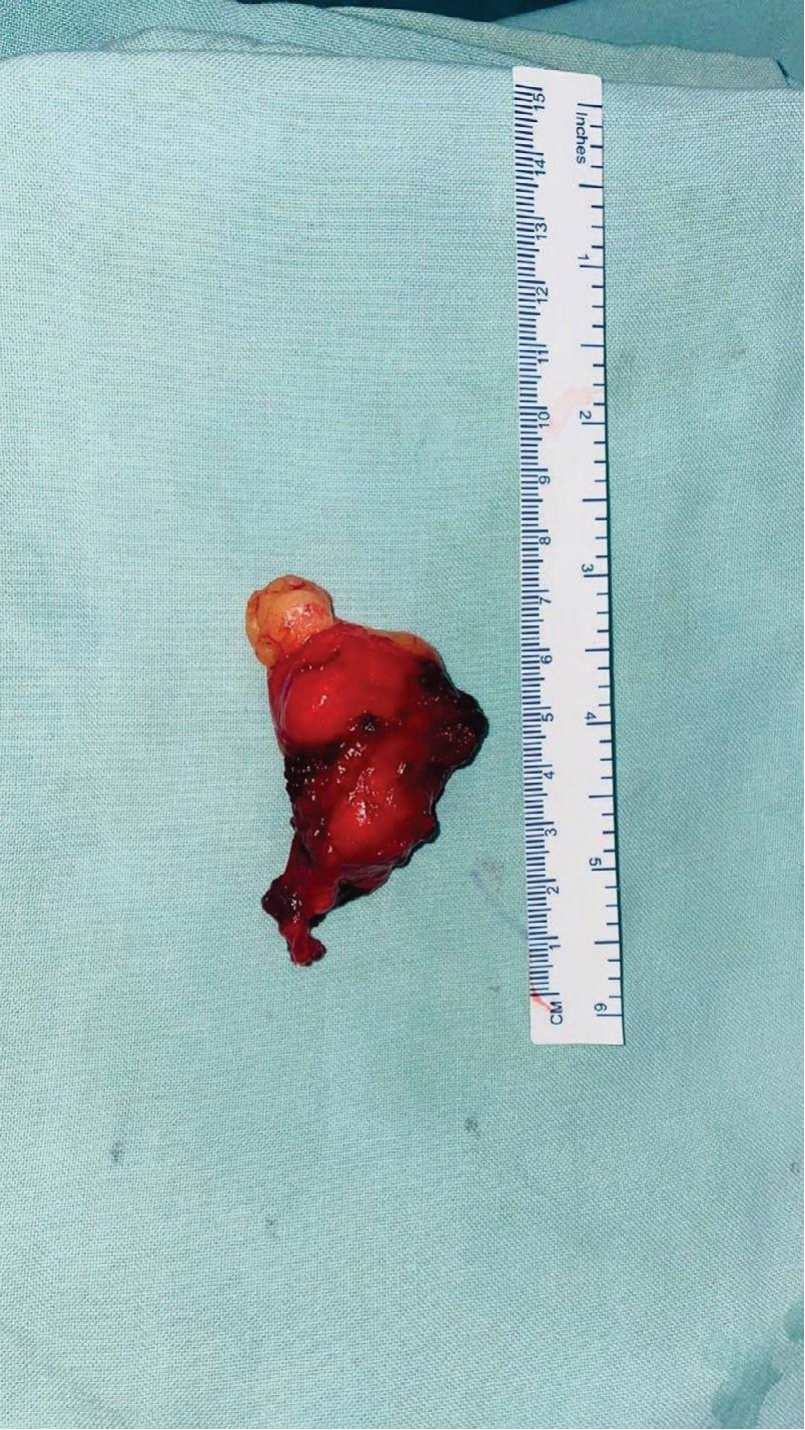
Lipoma excised from the forearm.

A dorsal approach between ECRL and brachioradialis was used to expose the PIN. A deep fatty mass was found compressing the nerve against the arcade of Frohse (Figure 1). After decompressing the arcade of Frohse, PIN was found to be severely compressed (Figure 2). A 8 × 5 cm mass was excised from the forearm (Figure 3) and sent for biopsy. Histopathological examination of the mass revealed lobules of adipocytes in between the muscle bundles separated by fibrocollagenous tissue with no atypical features. The features were compatible with intramuscular lipoma.

After the surgery, patient was advised physiotherapy and a dynamic cock up splint. But she only followed up for 2 weeks and was eventually lost to follow‐up. She showed up after 6 months post‐surgery.

**Figure 4:**
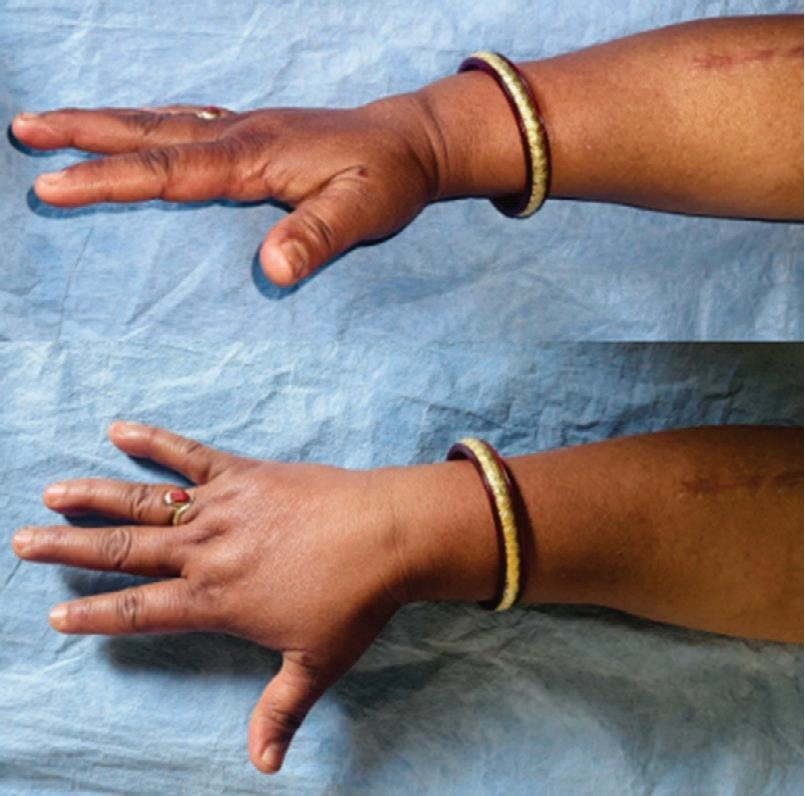
Thumb/fingers extension 6 months post‐surgery.

At 6 months post‐surgery, patient is recovering well and regained active finger and thumb extension (Figure 4).

## 3. Discussion

PIN compression can occur at multiple sites in the proximal forearm. Described etiologies include fibrous tissue anterior to the radiocapitellar joint, the radial recurrent vessels (known as the “leash of Henry”), the edge of the extensor carpi radialis brevis, the “arcade of Frohse,” and the distal edge of the supinator muscle [2]. The arcade of Frohse is cited as the most common site of compression [4, 5]. While space‐occupying lesions in this region are a recognized cause of PIN palsy, lipomas represent a very rare etiology [5, 6]. To date, only around 30 cases have been reported worldwide, and to our knowledge, this is the first to be reported from Nepal [7].

As a pure motor nerve, PIN palsy typically presents with motor weakness of the finger and wrist extensors, as seen in our patient. However, sensory involvement has been reported, attributed to concomitant compression of the superficial radial nerve given their anatomical proximity [8].

When available, magnetic resonance imaging (MRI) is the investigation of choice for diagnosing space‐occupying lesions. Ultrasound has also proven useful in diagnosing deep lipomas [8]. However, in settings where advanced imaging is not always accessible, a careful clinical examination remains the mainstay of diagnosis and is crucial for treatment planning. As with all nerve compression syndromes, the principle of “the earlier the decompression, the better the outcome” holds true for PIN palsy [9]. But, in our patient who presented with a 1‐year history of symptoms, decompression was still offered as a viable option. There has been report of recovery when decompression was done even after 18 months of symptom onset [10]. A recent meta‐analysis supports this approach, suggesting that a symptom duration greater than 18 months is a more significant negative predictor of motor recovery [7]. The same meta‐analysis reported a mean time to full motor recovery of 9.7 months (range: 1.5–60 months) [7]. Our patient achieved near‐normal recovery within 6 months’ post‐surgery. In cases of PIN compression, especially those due to a space‐occupying lesion, early surgical decompression is the mainstay of treatment [11]. It would have been very interesting to determine when recognizable recovery began and how it progressed; however, as our patient was lost to follow‐up, we were unable to assess this. The inability to attain proper imaging due to financial constrain is another limitation of this report.

## 4. Conclusion

This case of PIN compression by a lipoma illustrates the necessity for consideration of atypical causes in patients presenting with nerve compression symptoms. This also emphasizes the importance of detailed history and thorough clinical evaluation for the diagnosis of such cases especially when we have limited access to diagnostic imaging.

## Funding

No funding was received for this manuscript.

## Consent

Written consent has been taken from the patient for publication of the case. Being a case report, IRB clearance was not required for this article. This article has been written following CARE guideline.

## Conflicts of Interest

The authors declare no conflicts of interest.

## Supporting information


**Supporting Information** Additional supporting information can be found online in the Supporting Information section. Patient consent form, CARE checklist.

## Data Availability

Data sharing not applicable to this article as no datasets were generated or analyzed during the current study.
